# Human Mesenchymal Stromal Cells Derived from Different Tissues Show Similar Profiles of c-ErbB Receptor Family Expression at the mRNA and Protein Levels

**DOI:** 10.3390/ijms26157201

**Published:** 2025-07-25

**Authors:** Vera Kosheverova, Marianna Kharchenko, Rimma Kamentseva, Michael Kotov, Alexander Schwarz, Ivan Kuneev, Anastasia Kotova, Natella Enukashvily, Elena Kornilova

**Affiliations:** 1 Laboratory of Intracellular Membranes Dynamics, Institute of Cytology of the Russian Academy of Sciences, Tikhoretsky Ave. 4, Saint-Petersburg 194064, Russia; kosheverova_vera@incras.ru (V.K.); kharchenko_m@incras.ru (M.K.); lenkor@incras.ru (E.K.); 2 Institute of Biomedical Systems and Biotechnology, Peter the Great Saint-Petersburg Polytechnic University, Polytechnicheskaya, 29, Saint-Petersburg 195251, Russia; 3 Laboratory of Molecular Mechanisms of Neural Interactions, Sechenov Institute of Evolutionary Physiology and Biochemistry of the Russian Academy of Sciences, Saint-Petersburg 194223, Russia; aleksandr.pavlovich.schwarz@gmail.com; 4 Intracellular Signaling Laboratory, Institute of Cytology of the Russian Academy of Sciences, Tikhoretsky Ave. 4, Saint-Petersburg 194064, Russia; 5 Laboratory of the Non-Coding DNA Study, Institute of Cytology of the Russian Academy of Sciences, Tikhoretsky Ave. 4, Saint-Petersburg 194064, Russia

**Keywords:** human mesenchymal stromal cells, c-ErbB receptors, mRNA and protein expression, lysosomal degradation

## Abstract

The c-ErbB receptor family is a fundamental cell signaling system that regulates cell proliferation, motility, apoptosis, differentiation, and other key cellular functions. Overexpressed and mutated in some tumors, c-ErbB receptors play a pivotal role in their progression but are also present in many non-malignant cells, including those that are promising from the point of view of regenerative medicine, such as mesenchymal stromal cells (MSCs). The role of c-ErbB receptors in these cells is not clearly understood, and the data on their expression are sporadic. Therefore, the systemic characterization of c-ErbB receptor family expression in MSCs from a wide range of tissues is of high priority. Here, using RT-qPCR and Western blotting analysis, we evaluated the c-ErbB receptors expression pattern at the mRNA and protein levels in human MSCs isolated from six different tissues. We found that MSCs possess considerable *EGFR* and *HER2* mRNA levels comparable to those in some malignant cells while showing trace *HER3* and *HER4* expression. However, EGFR but not HER2 was detected in MSCs at the protein level. We also show that the absence of HER2 protein is not associated with its rapid lysosomal degradation. We conclude that c-ErbB signaling in human MSCs is exclusively mediated by EGFR.

## 1. Introduction

The epidermal growth factor receptor (EGFR/c-ErbB1/HER1) belongs to the c-ErbB family of transmembrane tyrosine kinase receptors, named after the discovery that the avian erythroblastosis tumor virus, v-ErbB, carries a mutated version of the human cellular EGF receptor (c-ErbB). Together with three other receptors of the c-ErbB family (c-ErbB2/HER2/neu; c-ErbB3/HER3; c-ErbB4/HER4) and more than 10 growth factor ligands, they form the c-ErbB signaling system. Proliferation and differentiation, cell motility, and apoptosis, as well as embryogenesis, are all regulated by c-ErbB family receptors, which are found in various cell types. Overexpression of the c-ErbB receptor family is common in tumors, making it a key diagnostic marker and pharmacological target in anticancer therapy [1,2,3].

In general, cell surface c-ErbB receptors are activated upon binding to the specific ligands. Ligand binding leads to receptors’ dimerization and subsequent activation of their intrinsic tyrosine kinase domains which cross-phosphorylate specific tyrosine residues in the cytoplasmic receptor domain, the sites for the recruitment of a set of effectors, thereby switching on a number of signaling pathways. Signaling from c-ErbB receptors is determined in many ways. Although the receptors demonstrate a high degree of structural similarity, the difference between the members’ sequences results in some specificity in the ligands and intracellular partners with which they can interact. Signal specificity also depends on whether the receptor forms homo- or heterodimers with other members of the c-ErbB family. For example, EGFR and HER4 can dimerize with themselves and other family members, whereas the orphan receptor HER2 mainly forms heterodimers with other ligand-activated family members. The composition of c-ErbB receptors expressed by a cell varies from one cell type to another [4], and the levels of specific c-ErbB proteins determine the predominant forms of homo- and heterodimers, ensuring the plasticity of the physiological cells response.

The amplification of c-ErbB genes and the high protein levels of c-ErbB receptors correlate with malignancy [5] and are therefore often considered as negative prognostic factors in the clinic. At the same time, c-ErbB receptors are expressed in many non-malignant tissues. Namely, *EGFR* expression was found in tissues of epithelial, mesenchymal, and neuronal origin [6]. *HER2* was reported to be expressed in epithelial cells of the gastrointestinal, respiratory, reproductive, and urinary tracts as well as in skin, breast, and placenta. HER2 protein and its transcript expression were shown to be higher in the fetal tissues than in the corresponding normal adult tissues [7]. *HER3* expression has been reported in normal tissues such as the human liver, kidney, brain, normal keratinocytes, and glandular epithelial tissues. However, *HER3* expression was not detected in fibroblasts, skeletal muscle, or lymphoid cells [8,9,10]. *HER4* was shown to be expressed in the heart, nervous system, and endocrine system [3].

Mesenchymal stromal cells (MSCs) are tissue-specific fibroblast-like cells with a high proliferative potential that show no signs of transformation. According to the latest report of the International Society for Cell and Gene therapy (ISCT), MSCs could be characterized as a heterogeneous population of cells isolated from a number of different tissues that should be positive for CD73, CD90, and CD105 and negative for CD45 [11]. The use of MSCs in the field of regenerative medicine seems to be a very promising approach [12] as their administration to the organism did not lead to tumorigenesis in mouse tumor xenograft models [13]. There are numerous registered clinical trials studying the effectiveness and safety of MSC-based therapy. Phase 3 and 4 trials use MSCs to treat osteoarthritis [14,15], limb ischemia [16], cirrhosis [17], heart failure [18], and other diseases. The current state of the field is regularly reviewed in profile articles [19,20]. Researchers are also focusing on the MSC secretome in regenerative medicine as a potential cell-free therapy method [21]. No serious side effects of MSC application were registered [22]. Recently, the FDA approved the first MSC-based therapy, Ryoncil (remestemcel-L-rknd), developed by Mesoblast Inc. (New York, NY, USA). This therapy uses ex vivo cultured bone marrow mesenchymal stromal cells (MSCs) from healthy adult human donors for treating the steroid-refractory acute graft-versus-host disease (SR-aGVHD) in pediatric patients [23]. In addition, MSCs could potentially serve as an experimental model of normal, non-transformed cells as finding differences in signal transduction in transformed and normal cells is of the top priority in cell science studies. The above points to the crucial need for a more in-depth understanding of the signaling pathways that are implemented in MSCs.

Expression of some c-ErbB receptors was reported in several types of human MSCs, including those derived from bone marrow [24,25], Wharton’s jelly [26], endometrium [27], adipose tissue [28], and periodontal ligament [29]. However, to our knowledge, systemic studies of c-ErbB receptors expression in MSCs are absent to date, and the involvement of the EGF receptor system in the regulation of MSC activity is often assessed by the dependence of these cells on certain ligands of these receptors. c-ErbB signaling has been reported to regulate MSC proliferation, migration, and its paracrine activity. In particular, c-ErbB receptor ligands, EGF and HB-EGF, have been shown to stimulate the proliferation of the human bone marrow MSCs [30,31,32]. These ligands as well as TGF-α were reported to be involved in the regulation of the proliferation and/or decidualization of human endometrial MSCs [33,34,35]. EGF could also stimulate the motility of bone marrow MSCs and induce the secretion of vascular endothelial growth factor (VEGF), hepatocyte growth factor (HGF), and other growth factors and cytokines in these cells [25,36]. Note that all aforementioned ligands specifically bind EGFR but are different in their affinity and the effect on EGFR intracellular behavior [33,37].

Taking into account the above-mentioned circumstances, it is important to describe the c-ErbB expression profile of MSCs in order to determine whether this is typical for MSCs as such or if it depends on their tissue origin. In the present work we have investigated the expression levels of c-ErbB receptors in a panel of human MSCs, isolated from six different tissues (uterine endometrium, Wharton’s jelly of the umbilical cord, dental pulp of deciduous teeth, periodontal ligament, fetal bone marrow, and adipose tissue) in comparison with the levels in six human malignant cell lines by RT-qPCR and Western blotting analysis. Such double control is necessary because signaling depends on a protein, and the effective receptor level for the EGFR signaling system may be regulated not only by gene expression but also by the regulation of translation or degradation upon ligand-stimulated endocytosis.

## 2. Results

### 2.1. c-ErbB Receptors mRNA Expression in Human MSCs Derived from Different Tissues

**Figure 1:**
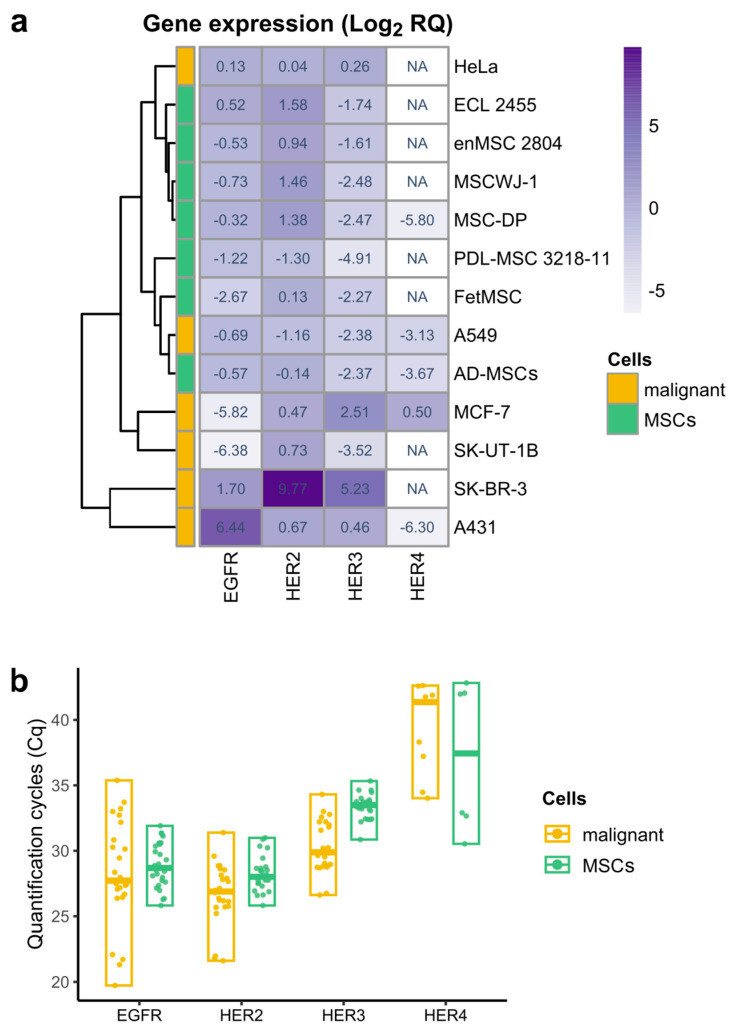
Heatmap with hierarchical clustering representing relative expression of *EGFR*, *HER2*, *HER3*, and *HER4* genes in six lines of human malignant cells (yellow) and seven types of human mesenchymal stromal cells (green), derived from different tissues. (**a**) Relative gene expression was obtained using real-time qPCR and calculated with the 2^−ΔΔCt^ method normalized against the geometric mean of the three reference genes (*YWHAZ*, *POP4*, and *EIF2B1*) and HeLa (for *EGFR*, *HER2*, and *HER3*) or MCF-7 (for *HER4*) expression levels. The meaning in each heatmap cell is the median of the log_2_-transformed relative gene expression values of the biological samples of the indicated cell type. The heatmap cell color corresponds to median value and changes from light (the lowest relative gene expression value) to deep purple (the highest relative gene expression value). A white color of the cell and NA abbreviation indicates that no expression was registered. Hierarchical clustering was performed using the *pheatmap* R package (package ver. 1.0.12, R ver. 4.2.3). (**b**) Quantification cycles (Cq) values of *EGFR*, *HER2*, *HER3*, and *HER4* receptors in malignant (yellow) and mesenchymal stromal cells (MSCs, green). Data are presented as median and range. Biological samples with no detected expression are not shown.

First, we aimed to reveal what members of the c-ErbB receptor family are expressed in the human MSCs at mRNA level and if there are substantial differences in this expression depending on tissue origin. To answer this question, we performed RT-qPCR analysis of MSCs, derived from human uterine endometrium (enMSC 2804; ECL 2455), Wharton’s jelly of umbilical cord (MSCWJ-1), dental pulp from deciduous teeth (MSC-DP), periodontal ligament (PDL-MSC 3218000011, in figures the number is shortened to 3218-11), fetal bone marrow (FetMSC), and adipose tissue (AD-MSCs). We also included six well-described human malignant cell lines to serve as references for c-ErbB receptor family mRNA levels (HeLa, MCF-7, SK-BR-3, A549, SK-UT-1B, and A431). The overall RT-qPCR data are presented in Figure 1 as the heatmap for ease of comprehension, while the detailed graphs showing group sizes and statistical differences are shown in Figure S1.

We found that all examined MSC lines were characterized by the considerable mRNA levels of *EGFR* (*HER1*) and *HER2* receptors which are comparable to their levels in some malignant cells (Figure 1b). Indeed, *EGFR* mRNA expression levels in MSCs do not show statistically significant differences with the malignant HeLa and A549 cell lines (Figure S1). *HER2* mRNA is expressed at relatively high levels in the majority of MSCs and malignant cells (Figure 1b).

Within the MSC group, *HER2* mRNA levels were also quite similar, except for periodontal ligament MSCs, which showed reduced expression compared to MSC-DP, MSCWJ-1, and enMSC 2804 cells (Figure S1). Between MSC and malignant cell groups, changes were detected only with malignant SK-BR3 cell line, known to overexpress *HER2* [38] and A549 cell line, where *HER2* expression was decreased (Figure S1). Unlike *EGFR* and *HER2*, *HER3* and *HER4* show little or no expression in all MSCs examined (Figure 1b). Although we identified *HER3* expression in MSCs with the use of *HER3*-specific FAM-BHQ1-labeled probe, this expression was near the limit of the sensitivity of the method. As for *HER4*, its trace mRNA levels were detected only in AD-MSCs and MSC-DP cells (Figure 1a).

Thus, in our cell panel the essential feature of proliferating MSCs derived from different tissues turned out to be its c-ErbB family expression profile similarity. In contrast, malignant cell lines are characterized by much more pronounced variability in c-ErbB family expression, which is clearly demonstrated by the heatmap (Figure 1a).

### 2.2. EGFR but Not HER2 Protein Is Revealed in MSCs

We have shown that *EGFR* and *HER2* mRNA are expressed at considerable levels in human MSCs derived from different tissues. Next, we have checked if these receptors are detectable in MSCs at the protein level.

**Figure 2:**
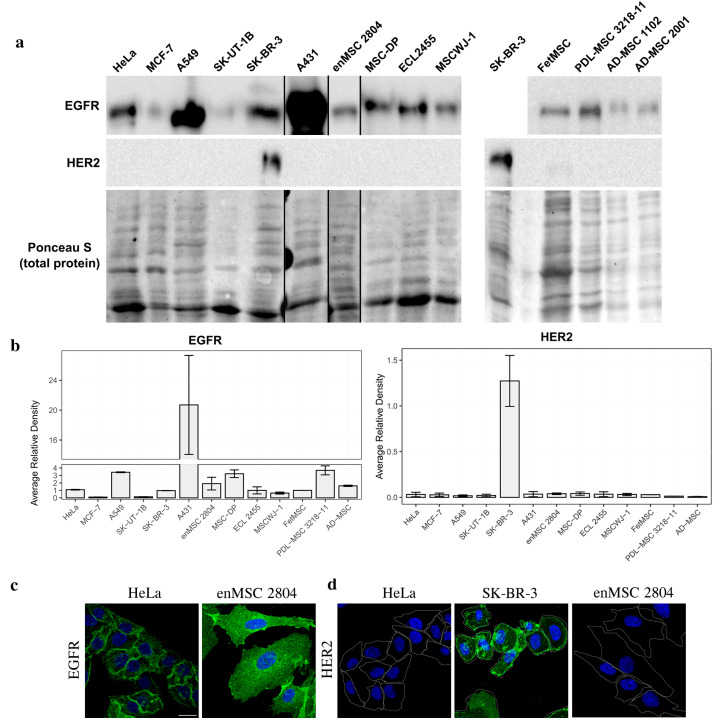
EGFR but not HER2 protein expression is detected in human MSCs. (**a**) Total cell lysates of the human malignant cells and MSCs of different origin were separated by SDS-PAGE and processed for Western blotting. Blots were stained with anti-EGFR antibodies (upper line) and anti-HER2 antibodies (middle line). Nitrocellulose membrane was stained with Ponceau S solution for total protein and used as the loading control (lower line). (**b**) The Western blots were quantified and normalized against SK-BR-3. The densitometry data are presented as the means ± s.e. (**c**,**d**)—immunofluorescence of EGFR (**c**) and HER2 (**d**) in HeLa and enMSC 2804 cells. HeLa, enMSC 2804, and SK-BR-3 were fixed, permeabilized, and stained with anti-EGFR or anti-HER2 antibodies followed by GAR-Alexa Fluor 488 secondary antibodies staining (green). The cell nuclei were visualized using Hoechst 33285 (blue). Cell borders are highlighted in white. Scale bar: 20 µm.

We performed Western blot analysis on total cell lysates from MSCs and malignant cell lines and stained the blots with anti-EGFR and anti-HER2 antibodies (Figure 2a). EGFR was detected in all MSCs tested, and its level varied to a lesser extent than within the group of malignant cell lines. When comparing the amount of EGFR protein in MSCs and malignant cells, the closest malignant cell line in this case turned out to be HeLa (Figure 2). We have also shown that EGFR protein in endometrial MSC line 2804 could also be detected by immunofluorescence staining with comparable visibility as in HeLa cells (Figure 2b). EGFR protein levels as well as *EGFR* mRNA levels were found to be highly variable in the group of malignant cells. EGFR protein was most abundant in the A431 cell line, while EGFR protein was detected at low levels in MCF-7 and SK-UT-1B cells (Figure 2), which is consistent with the data from other laboratories [39,40].

On the contrary, when the amount of HER2 protein was analyzed both by Western blotting and immunofluorescence, no positive staining could be detected in all MSC types examined (Figure 2a,b). Among malignant cells, SK-BR-3 was the only HER2-positive cell line (Figure 2a,b). It should be noted that we initially used the SK-BR-3 cell line as the HER2-positive control, as SK-BR-3 is well known to have high levels of HER2 protein [38]. Thus, while *EGFR* and *HER2* mRNA levels are quite high in MSCs, at the protein level only EGFR, but not HER2 protein, could be detected in these cells.

### 2.3. The Failure of HER2 Protein Detection in MSCs Is Not Associated with Its Rapid Lysosomal Degradation

**Figure 3:**
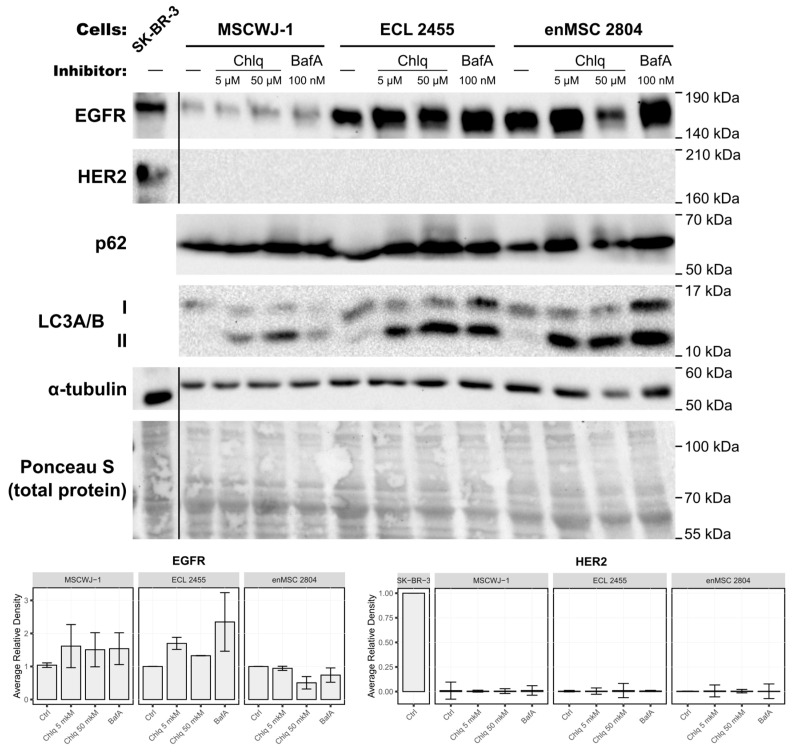
The absence of HER2 expression at the protein level is not associated with its rapid degradation via the lysosomal pathway. Three different types of the human MSCs (MSCWJ-1, ECL 2455, and enMSC 2804) were subjected to 24 h treatment with lysosomal inhibitors Chloroquine (Chlq, C = 5 µM; 50 µM), or Bafilomycin A1 (BafA, 100 nM). Total cell lysates of these cells and the malignant SK-BR-3 cell line (served as HER2-positive control) were separated by SDS-PAGE and processed for Western blotting. Blots were stained with anti-EGFR, anti-HER2, anti-p62 (Abcam Inc., USA), anti-LC3A/B, and anti-alpha tubulin antibodies. The nitrocellulose membrane was stained with Ponceau S solution for total protein analysis and used as a loading control. The EGFR and HER2 Western blots were quantified and normalized against each line control cells (Ctrl) or SK-BR-3 in the case of HER2. Relative optical densities are presented as the means ± s.e of three independent experiments.

c-ErbB family receptors is known to undergo down-regulation mainly via the endosomal–lysosomal degradative pathway [41]. Thus, we should not exclude the possibility that HER2 protein in MSCs could not be detected because it is degraded in lysosomes shortly after its synthesis. To check this assumption, we incubated three types of MSCs (MSCWJ-1, ECL 2455, and enMSC 2804) with lysosomal inhibitors Chloroquine (Chlq) and Bafilomycin A1 (BafA), which were previously shown to block EGFR degradation by inhibiting canonical lysosomal pathway [42] and thus should inhibit possible HER2 degradation in lysosomes (Figure 3). It should be noted that Chloroquine and Bafilomycin A1 are known to stimulate the formation of autophagosomes [43,44]; thus, the observed accumulation of autophagosome markers in experiments using these inhibitors does not reflect the basal level of autophagy in these cells. At the same time both reagents are known to increase the intralysosomal pH by different mechanisms, thus inhibiting lysosomal proteolytic activity [44].

We incubated MSCs for 24 h in the complete medium containing Chloroquine at final concentrations of 5 µM or 50 µM or the V-ATPase’s inhibitor Bafilomycin A1 at a concentration of 100 nM.

To test the inhibitors’ efficiency, the blots were stained with anti-p62 and anti-LC3A/B antibodies. Since both Chloroquine and Bafilomycin A1 induce autophagosome formation and block cargo degradation within lysosomes [42], p62 and LC3A/B (microtubule-associated protein light chain 3) serve as the classical markers for monitoring the activity of these inhibitors. When autophagy is induced, the cytosolic form of LC3 (LC3-I) conjugates with phosphatidylethanolamine of nascent autophagosome membrane, and thus is called lipidated or membrane-bound LC3 (LC3-II). After Chloroquine and Bafilomycin A1 treatment, the amount of LC3-II should increase. p62 (also known as SQSTM1) binds both to ubiquitinated cargo of autophagosomes and to LC3, thus serving as the adaptor protein that is itself degraded in autolysosomes [45]. Therefore, the p62 amount should increase in cells when lysosome functioning is blocked.

Indeed, after treatment with inhibitors, the amount of p62 increased in inhibitor-treated samples (Figure 3 and Figure S2), indicating the efficient block of cargo degradation in lysosomes. Chloroquine and Bafilomycin A1 treatment also caused a pronounced increase in the amount of LC3-II (lower band).

If our hypothesis was confirmed, then HER2 staining would be observed by Western blotting in inhibitor-treated cell samples. However, no HER2-positive bands were detected in these inhibitor-treated samples (Figure 3). At the same time, EGFR staining was detected in inhibitor-treated and untreated samples with a tendency to increase in Chloroquine- and Bafilomycin A1-treated samples (Figure 3).

Thus, we conclude that though Chloroquine and Bafilomycin A1 treatment was effective and inhibited cargo degradation by lysosomes, it did not lead to the appearance of detectable HER2 protein in MSC samples.

## 3. Discussion

In this article we have characterized the expression profile of c-ErbB receptors at the mRNA and protein level in a panel of human MSCs derived from a variety of tissues and have compared it with the profile in several cell lines obtained from tumors. We found that at the mRNA level all MSC types express *EGFR* and *HER2* at considerable levels while showing trace expression of *HER3* and *HER4*, with the latter being detected only in MSC-DP and AD-MSCs. On the contrary, in malignant cells c-ErbB receptor family expression was more heterogeneous.

Although we could not find any systemic analyses in the literature comparing c-ErbB receptor family expression in MSCs from different tissues in one study, there are some data on the expression of certain members of this family in human MSCs. Namely, *EGFR* mRNA was found in the human bone marrow MSCs [24,41], human endometrial MSCs [27], and in Wharton’s jelly-derived MSCs [26]. At the protein level EGFR was detected in human adipose-derived MSCs [28] and periodontal ligament stromal cells [29]. Thus, according to the literature and our data, EGFR/HER1 may be abundantly present in human MSCs at both the mRNA and protein levels. Increased level of EGFR expression is often found in some tumors and is believed to correlate with enhanced proliferation. However, MSCs expressing comparable levels of EGFR did not demonstrate any signs of transformation. This indicates that overexpression of EGFR is not ultimately bound to tumorigenesis, and there are mechanisms preventing uncontrolled growth in this case.

Some tumorigenesis mechanisms may be related to the presence of not only EGFR, but also other members of the family capable of forming heterodimers that stimulate a stronger proliferative signal. Indeed, HER2 expression is generally studied because of its association with tumorigenesis as its amplification/overexpression drives malignant transformation [46]. Regarding HER2 expression in normal human MSCs, it has been reported that *HER2* mRNA is present in human bone marrow MSCs [25]. We also found its expression in all MSCs of our panel but failed to detect HER2 protein. The absence of HER2 protein cannot be explained by constitutive internalization and lysosomal degradation as it was shown by our experiments with inhibitors of degradative pathway.

To our knowledge, there are no data in the literature on *HER3* and *HER4* expression in human MSCs, but their expression has been found in some normal non-malignant tissues, as well in different types of tumors [47].

One of our interesting findings regarding the c-ErbB mRNA expression profile of MSCs from six different types of tissue is their relative homogeneity, as few statistically significant differences were detected within the MSC group. The malignant cell group showed a profound heterogeneity (Figure S1), which can be explained by the known common amplifications of c-ErbB receptors in cancer. We have previously shown that MSCs are more homogeneous compared to malignant cells in gene expression, not only for c-ErbB genes but also for housekeeping genes [48]. This property may be advantageous for the clinical application of MSCs.

When comparing c-ErbB mRNA expression levels between MSCs and malignant cells, HeLa is the closest malignant cell line because there are no significant differences in *EGFR* and *HER2* mRNA levels between HeLa and MSCs. The only difference is the increased *HER3* mRNA level in HeLa cells compared to periodontal MSCs (Figure S1). Thus, c-ErbB mRNA expression levels are comparable in MSCs and some cancer cell lines.

When we analyzed EGFR and HER2 expression at the protein level, we found that EGFR, but not HER2, was detected in MSCs (Figure 2). Among the malignant cells, the only HER2-positive cell line was SK-BR-3, which is derived from metastatic breast adenocarcinoma and is known to overexpress HER2 protein. Thus, there is some inconsistency between the relatively high levels of *HER2* mRNA and the absence of HER2 protein in MSCs and the majority of malignant cell lines.

This discrepancy between *HER2* mRNA and protein levels has been previously reported in the literature. The study by Ma et al. examined *HER2* mRNA and protein profiles in eighty gastric mucosal specimens obtained from patients with gastric precancer, gastric cancer, or no cancer pathology [49]. The authors showed that HER2 protein staining analyzed by immunohistochemistry was positive in 0% of cases in normal mucosal tissues, 10% in precancerous tissues, and 30% and 40% in early and advanced gastric cancer tissues, respectively. At the same time, *HER2* mRNA levels were the highest in normal tissues and decreased in the «precancer-cancer» line. The authors did not detect *HER2* gene amplification in any of 20 samples from patients with different grades of gastric cancer [49]. Although *HER2* gene amplification status usually correlates well with the HER2 protein levels, even in cancer samples with confirmed *HER2* gene amplification the protein may not always be detected. It was shown that among 331 breast cancer samples characterized as positive for *HER2* gene amplification by FISH, 13 samples (3.9%) were HER2-protein-negative as assessed by immunohistochemistry analysis [50]. Another recent study [51] evaluated the correlation between *HER2* mRNA levels measured by RNAscope and HER2 protein expression as assessed by PATHWAY and HercepTest IHC assays in tissue samples from patients with metastatic breast cancer. It was reported that while the mRNA levels correlated strongly with the IHC H-scores which reflect the amount of HER2 protein in samples, this correlation disappeared at the low IHC H-scores (0, 1+), when HER2 protein was not detected (0), or was detected at low levels (1+) [51]. Another group reported that while qPCR analysis was able to discriminate HER2-protein-positive gastroesophageal cancers with high IHC H-scores (IHC3+) from HER2-negative cancers (IHC0/1+) with 81.6% accuracy, it failed to discriminate HER2-equivocal samples from HER2-positive or -negative cancers as determined by in situ hybridization data [52]. Thus, mRNA levels determined by RT-qPCR cannot reliably predict HER2 protein levels in cases where the *HER2* gene is not amplified in both cancerous and normal non-malignant tissues.

The exact reasons for this discrepancy between *HER2* mRNA and protein levels are still not fully understood. It is well known that EGFR enters the canonical degradation pathway upon EGF binding followed by the receptor tyrosine kinase activation which promotes its ubiquitination by c-Cbl. HER2 is an orphan receptor, and its lysosomal degradation can be triggered by HER2 heterodimerization mostly with other members of c-ErbB family receptors (HER1, 3 and 4) [53]. However, it has been shown that in cancer cells, especially in cases when HER2 protein is overexpressed, its degradation is regulated by the mechanism different from the canonical one [54]. In such cancers, HER2 protein binds to a protein called FAM83A (Family with sequence similarity 83 member A) [55] and the chaperone Hsp90 [56], both of which are overexpressed in tumors [55,57]. FAM83A and Hsp90 inhibit HER2 ubiquitination, internalization into endosomes, and subsequent lysosomal degradation [55,56]. It was also demonstrated that the use of anti-HER2 antibodies and Hsp90 inhibitors allowed overcoming the HER2 degradation block [54]. On the contrary, in non-malignant mammary epithelial cells lines HC11 and 31E, HER2 was shown to undergo lysosomal degradation, which was stimulated upon EGF addition possibly due to formation of heterodimers with EGFR [58].

Here we aimed to check whether the absence of HER2 protein in MSCs could be a result of rapid HER2 degradation in these cells. Note that we addressed mainly constitutive degradation in the absence of exogenous ligands. Since the main pathway of transmembrane c-ErbB receptor degradation is the delivery to lysosomes, we used Chloroquine and Bafilomycin A1 as inhibitors that block lysosomal degradation in the cells. However, the inhibition of lysosomal function by these inhibitors for 24 h did not increase the HER2 protein levels in MSCs, which indicated the absence of constitutive internalization and final lysosomal degradation of this receptor.

Another pathway of protein degradation is mediated by proteasomes. Although this pathway predominantly regulates cytoplasmic protein levels, some studies have shown that proteasome inhibitors may prevent the degradation of EGFR [59,60,61]. However, an earlier study by our group [62] showed that this inhibition was associated with MG132-induced depletion of the free ubiquitin pool. Ubiquitin is necessary for the sorting of proteins from early to late endosomes by ESCRT-complexes [63]. When the free pool of ubiquitin is depleted, the level of EGFR ubiquitination mediated by the ubiquitin ligase c-Cbl decreases [62]. Using subcellular fractionation, we also demonstrated that the EGFR protein is delivered to lysosomes in its full-length form. Thus, proteasome inhibitors may affect the lysosomal pathway, which complicates the interpretation of experimental data. So, according to the current view, degradation of transmembrane receptors, including HER2, is not normally carried out by proteasomes [64]. Instead, during Endoplasmic Reticulum-Associated Degradation (ERAD) defective transmembrane proteins are degraded by proteasomes, but only after retrotranslocation to the cytosol [65]. Multiple targeted degradation therapy strategies are based on this paradigm [66].

Therefore, translational control mechanisms should be considered central to the regulation of HER2 protein maintenance. Indeed, such an inhibitory mechanism has already been reported for *HER2* translation. It was found that HER2 synthesis could be regulated by the upstream open reading frame (uORF), which is present in the 5′-untranslated region (UTR) of *HER2* mRNA [67]. This regulatory element generally contributes to translational suppression in most cells. However, in cancer, this uORF-dependent mechanism may be altered [68]. In particular, it has been shown that the 3′-UTR of the *HER2* mRNA can override the translation inhibition mediated by the *HER2* uORF in HER2-overexpressing breast cancer cells [69]. The authors proposed that this is associated with the increased expression of several RNA-binding proteins, including HuR and hnRNP C1/C2, in breast cancer cells.

Our data support the idea that translational suppression occurs in MSCs as it does in normal, non-transformed cells. However, to directly test this hypothesis, one should artificially override the translation suppression to increase the HER2 protein level. Several studies have explored methods for reducing HER2 level given its significance in treating HER2+ cancers [70,71]. Conversely, there is currently no proven approach to increase this protein’s level without directly overexpressing *HER2* with a plasmid. This could be achieved by overexpressing or modulating the activity of regulatory proteins involved in *HER2* translation, such as HuR, hnRNP C1/C2 [69], Hsp90 [54], or ABL [72]. However, these factors regulate the expression of numerous other proteins, which would substantially alter the MSC proteome and potentially transform the cells.

Thus, we have demonstrated that the expression profiles of MSCs of different origins are remarkably homogeneous and characterized by high mRNA expression of *EGFR* and *HER2* as well as low expression of *HER3* and in some cases *HER4*. At the same time, at the protein level, EGFR seems to be the predominant member of the c-ErbB receptors in all cultured MSCs studied. Taking into account that HER2 is the preferred partner for HER3 and HER4 in heterodimerization, we suggest that in MSCs these receptors do not play a significant role, whereas the main control of signaling is carried out by EGF receptor homodimers and its ligands. This allows us to propose that MSCs may be the convenient in vitro model for studying normal EGFR signaling.

## 4. Materials and Methods

### 4.1. Cell Cultures

We used 6 human malignant cell lines and human mesenchymal stromal cells, obtained from 6 different tissues. The information regarding the tissue of origin, the type of tumor (in the case of malignant cells), and the source of the cells (source/cell culture collection from which the cells were obtained) are listed in Table 1. Characterization of the cells by Short Tandem Repeat (STR) profiling, MSC markers surface expression (in case of MSCs), etc., was performed by the corresponding source/cell culture collections. Details regarding the isolation and characterization of MSCs can be found in the publications referenced in Table 1. All cell lines tested negative for mycoplasma.

All MSCs used except those isolated from periodontal ligament (PDL-MSC 3218000011, in figures the number is shortened to 3218-11) were maintained in DMEM/F12 medium with phenol red (Cat. No. 31330-038, Gibco™, Life Technologies Ltd., Paisley, UK) supplemented with 10% fetal bovine serum (Cat. No. S181S, Biowest SAS, Nuaillé, France), GlutaMAX™ (Cat. No. 35050061, Gibco™, Life Technologies Ltd., Paisley, UK), and antibiotic/antimycotic solution (Cat. No. AAS-B, Capricorn Scientific GmbH, Düsseldorf, Germany). PDL-MSC 3218000011 cells were cultured in the DMEM medium with a glucose concentration of 1 g/L, supplemented with L-Glutamine, Sodium Pyruvate, and phenol red (Cat. No. DMEM-LPA, Capricorn Scientific GmbH, Düsseldorf, Germany) and containing the same components in the same concentrations (serum, GlutaMAX™, antibiotic/antimycotic solution) listed as above. The MSCs of the 7–17 passages were included in this study.

The HeLa, A549, SK-UT-1B, and A431 cells were maintained in DMEM medium containing 4.5 g/L glucose and phenol red (Cat. No. 11965092, Gibco™, Life Technologies Ltd., Paisley, UK) and supplemented with 10% fetal bovine serum, GlutaMAX™, and antibiotic/antimycotic solution. The MCF-7 and SKBR-3 cells were maintained in the same complete DMEM/F12-containing medium as the majority of MSCs. All cell lines were maintained at 37 °C in an atmosphere of 5% CO_2_.

### 4.2. Reagents

The lysosomotropic agent Chloroquine (Chlq, L10382, Molecular Probes™, Life Technologies Corp., Eugene, OR, USA), an inhibitor of endosomal acidification and endosome–lysosome fusion, was used at concentrations of 5 µM and 50 µM. The vacuolar vATPase inhibitor Bafilomycin A1 (BafA) was purchased from Sigma-Aldrich Inc., St. Louis, MO, USA, and used at a concentration of 100 nM. All inhibitors were dissolved in the complete medium and added to the cells for 24 h.

### 4.3. Western Blotting

For total cell lysate samples, cells grown to 70–90% confluence were resuspended on ice in lysis buffer containing 1% Triton X-100 (SERVA Electrophoresis GmbH, Heidelberg, Germany), 20 mM Tris-HCl, 150 mM NaCl, 1 mM EGTA, 1 mM EDTA, and Halt™ Protease and Phosphatase Inhibitor cocktail (Cat. No. 78442, Pierce Biotechnology Inc., Rockford, IL, USA). Cell lysates were homogenized by 10 passages through a 27 G needle. Supernatants were collected after the centrifugation at 4000 rpm for 5 min at 4 °C, mixed with Laemmli sample buffer and incubated at 99 °C for 5 min. Protein concentrations were determined by the Bradford protein assay using a Multiskan FC microplate photometer (Thermo Fisher Scientific Inc., Waltham, MA, USA).

SDS-PAGE electrophoresis was performed on 7.5% or 15% polyacrylamide gels according to Laemmli [79]. PageRuler™ Plus Prestained Protein Ladder (Cat. No. 26619, Thermo Fisher Scientific Inc., Waltham, MA, USA) was used to determine the molecular weight of the proteins. Proteins were wet-transferred to a 0.45 µm nitrocellulose membrane (Bio-Rad Laboratories Inc., Hercules, CA, USA) using the Mini Blot Module (Cat. No. B1000, Thermo Fisher Scientific Inc., Waltham, MA, USA). To detect protein bands, the nitrocellulose membrane was stained with Ponceau S (Sigma-Aldrich Inc., St. Louis, MO, USA).

Western blot analysis was performed according to the manufacturer’s standard protocols (Bio-Rad Laboratories Inc., USA). EGFR in samples was detected using rabbit anti-EGFR antibodies (Cat. No. 4267S, Cell Signaling Technology Inc., Danvers, MA, USA) at a dilution of 1:1000. We also used rabbit anti-HER2 antibodies (29D8, Cat. No. 2165, Cell Signaling Technology Inc., Danvers, MA, USA, dilution 1:1000) that were the generous gift of Dr. A. Daks (St. Petersburg, Russia). Rabbit anti-LC3A/B antibodies (D3U4C, Cat. No. 12741S, Cell Signaling Technology Inc., Danvers, MA, USA) were used at 1:1000 dilution. Mouse anti-p62 antibodies (1:1000, Cat. No. AB56416, Abcam Inc., Waltham, MA, USA) and rat anti-alpha tubulin antibodies (1:1500, Cat. No. MA1-80017, Invitrogen™, Thermo Fisher Scientific Inc., Eugene, OR, USA) were diluted in 3% non-fat milk containing TBS buffer (10 mM Tris; 74 mM NaCl, 0.01 M PBS) with the addition of 0.1% Tween (T-TBS). All other primary antibodies were diluted in 5% BSA-T-TBS solution. The nitrocellulose membranes were incubated with primary antibody solution overnight at 4 °C followed by its incubation with appropriate secondary antibodies for 1 h at room temperature (RT). We used goat anti-rabbit, goat anti-mouse (Cat. No. 7074 and 7076, Cell Signaling Technology Inc., Danvers, MA, USA, dilution 1:2000), or rabbit-anti-rat (1:10,000, Cat. No. SAA544Ra09, CloudClone Corp., Wuhan, China) HRP-conjugated secondary antibodies diluted in 5% BSA-T-TBS solution.

The chemiluminescence signal was detected with ECL SuperSignal™ West Femto Maximum Sensitivity Substrate (Cat. No. 34096, Pierce Biotechnology Inc., Rockford, IL, USA) using the ChemiDoc MP Imaging System (Bio-Rad Laboratories Inc., Hercules, CA, USA) and analyzed with the ImageLab software (version 6.0.1, build 34, Bio-Rad Laboratories Inc., Hercules, CA, USA).

### 4.4. Immunofluorescence and Laser Scanning Confocal Microscopy

Cells were grown to an approximately 70% monolayer on coverslips on the day of the experiment, fixed with 4% formaldehyde for 15 min at RT, washed with PBS, and permeabilized with 0.5% Triton X-100 for 15 min at RT. To prevent non-specific antibody binding, samples were incubated in 1% BSA for 30 min at RT. Cells were incubated with rabbit anti-EGFR (1:100, Cat. No. 4267S, Cell Signaling Technology Inc., Danvers, MA, USA) or rabbit anti-HER2 (1:200, 29D8, Cat. No. 2165, Cell Signaling Technology Inc., Danvers, MA, USA) antibodies in 1% BSA-PBS. After washing with PBS, the samples were incubated with secondary antibodies GAR-Alexa Fluor 488 (1:200, 20 min, 37 °C, Cat. No. A11008, Invitrogen™, Life Technologies Corp., Eugene, OR, USA), and the cell nuclei were visualized with Hoechst 33285 (1:500, Servicebio Technology Co., Ltd., Wuhan, China). The samples were mounted in 0.2 M DABCO (1,4-diazabicyclo(2.2.2)octane, Sigma-Aldrich Inc., St. Louis, MO, USA) glycerol-containing media. Fluorescence images were captured using the confocal laser scanning microscope Olympus FluoView3000 (Olympus Corp., Tokyo, Japan) with a 40×/1.3 objective and 405 and 488 nm lasers.

### 4.5. RNA Extraction and cDNA Synthesis

Total RNA was extracted from cells using an RNA Solo Kit according to the manufacturer’s instructions (Cat. No. BC034S, Evrogen JSC, Moscow, Russia). Three to seven biological samples were analyzed for one cell line/MSCs of one origin. Each biological sample included in analysis corresponded to a specific cell line grown on a single cell flask. The RNA concentration (260 nm absorbance) and purity (260/280 and 260/230 absorbance ratios) were measured in the obtained samples using a NanoDrop One Microvolume UV-Vis Spectrophotometer (Thermo Fisher Scientific Inc., Waltham, MA, USA).

Complementary DNA (cDNA) was synthesized from 800 ng of total RNA in a final volume of 20 μL using an MMLV RT kit (Cat. No. SK021, Evrogen JSC, Moscow, Russia) according to the manufacturer’s instructions. Reverse transcription was performed using 10 μM of oligo(dT) and random decamer primers in equal concentrations and 100 units of MMLV reverse transcriptase. The resulting mixture was incubated at 40 °C for 45 min for reverse transcription and then at 70 °C for 10 min for enzyme inactivation. cDNA was stored at −80 °C and diluted 10-fold in RNAse-free water before performing the qPCR.

### 4.6. Quantitative Polymerase Chain Reaction (qPCR)

qPCR was performed in a total volume of 6 μL with 0.8 μL of the cDNA sample using 5× qPCRmix-HS SYBR (Cat. No. PK147L, Evrogen JSC, Moscow, Russia) or, in the case of HER3-specific primers with FAM-BHQ1-labeled probe, 5× qPCRmix-HS (Cat. No. PK145L, Evrogen JSC, Moscow, Russia) that were used at a final concentration of 1×. Oligonucleotides were purchased from Evrogen JSC, Moscow, Russia. The amplification was performed on a CFX384 Touch RealTime PCR Detection System (Bio-Rad Laboratories Inc., Hercules, CA, USA) and included the hotstart step (95 °C for 3 min); 39 cycles of denaturation (95 °C for 5 s); and an annealing/elongation step (62 °C for 10 s) followed by the fluorescence plate reading. At the end of the amplification, a melting curve analysis was performed at 62–95 °C. All reactions included four technical replicates of each biological sample with no reverse transcription (NRT) and no template (NTC) controls.

The specific primer sequences are summarized in Table 2. We used 200 nM of specific forward and reverse primers for the *YWHAZ* gene, 300 nM for the *EGFR*, *HER2*, and *HER3* genes, 400 nM for the *POP4*, *EIF2B1*, and *HER4* gene, and 150 nM for the *HER3*-specific FAM-BHQ1-labeled probe.

Primers were previously designed in our laboratory [48] using the primer design tool Primer-BLAST (https://www.ncbi.nlm.nih.gov/tools/primer-blast/, accessed on 22 July 2025) or were obtained from previous publications [80,81]. Using serial dilutions, the primer efficiencies in the range of 93–112% were demonstrated.

Three most stable reference genes (*EIF2B1*, *YWHAZ*, and *POP4*) for normalization of expression genes of interest were selected from 8 candidate housekeeping genes according to a comprehensive ranking obtained using the RefFinder online tool (https://www.ciidirsinaloa.com.mx/RefFinder-master/, accessed on 22 July 2025) which is based on the GeNorm [82], NormFinder [83], BestKeeper [84], and comparative deltaCT [85] algorithms. The reference gene selection for the broad panel of malignant cell lines and MSCs was previously described in detail [48]. The validity of the previously selected reference genes (*EIF2B1*, *YWHAZ*, and *POP4*) was also confirmed for the three additional MSC types (FetMSC, PDL-MSC 3218000011, and AD-MSCs). The relative expressions of *EGFR*, *HER2*, *HER3*, and *HER4* were calculated using the 2^−ΔΔCt^ method and normalized to the geometric mean of the three selected reference genes (*EIF2B1*, *YWHAZ*, and *POP4*). The obtained data were analyzed using Bio-Rad CFX Maestro 2.3 software (version 5.3.022.1030, Bio-Rad Laboratories Inc., Hercules, CA, USA).

### 4.7. Statistical Analysis

Statistical analysis was performed using GraphPad Prism 9 (ver. 9.5.1.733, GraphPad Software Inc., San Diego, CA, USA). Differences in *EGFR*, *HER2*, *HER3*, and *HER4* expression between groups (cell types) were compared using the non-parametric Kruskal–Wallis H-test with the post hoc Dunn’s test and false discovery rate (FDR) correction for multiple comparisons using the two-stage step-up method of Benjamini, Krieger, and Yekutieli (Q = 0.01). Statistical significance was set at *p* < 0.05.

## Figures and Tables

**Table 1:** Cell types analyzed in this study and their brief characteristics and sources.

Cells	Tissue of Origin	Disease	Source (Obtained from)
**human malignant cells**			
HeLa	Uterus; Cervix	Adenocarcinoma	Vertebrate cell culture collection (Institute of Cytology RAS, St. Petersburg, Russia)
MCF-7	Breast; Mammary gland	Adenocarcinoma	Vertebrate cell culture collection (Institute of Cytology RAS, St. Petersburg, Russia)
A549	Lung	Carcinoma	Vertebrate cell culture collection (Institute of Cytology RAS, St. Petersburg, Russia)
SK-UT-1B	Uterus; Endometrium	Leiomyosarcoma	Vertebrate cell culture collection (Institute of Cytology RAS, St. Petersburg, Russia)
A431	Skin; Epidermis	Epidermoid carcinoma	Vertebrate cell culture collection (Institute of Cytology RAS, St. Petersburg, Russia)
SK-BR-3	Breast; Mammary gland	Adenocarcinoma	Generous gift from A. Daks, Institute of Cytology RAS, St. Petersburg, Russia
**human mesenchymal stromal cells**			
enMSC 2804	Uterus; desquamated (shedding) endometrium in menstrual blood	no	Vertebrate cell culture collection (Institute of Cytology RAS, St. Petersburg, Russia) [73]
ECL 2455	Uterus; endometrium, biopsy	no	Vertebrate cell culture collection (Institute of Cytology RAS, St. Petersburg, Russia) [74]
MSCWJ-1	Wharton’s jelly of umbilical cord	no	Vertebrate cell culture collection (Institute of Cytology RAS, St. Petersburg, Russia) [75]
MSC-DP	Dental pulp from deciduous (baby) teeth, 6-month-old baby	no	Vertebrate cell culture collection (Institute of Cytology RAS, St. Petersburg, Russia) [76]
FetMSC	Bone marrow of 5–6-week-old embryo	no	Vertebrate cell culture collection (Institute of Cytology RAS, St. Petersburg, Russia) [77]
PDL-MSC 3218000011	Periodontal ligament	no	Stem Cell Bank Pokrovsky, LLC, St. Petersburg, Russia [78]
AD-MSC AC110	Adipose-derived mesenchymal stromal cells	no	Vertebrate cell culture collection (Institute of Cytology RAS, St. Petersburg, Russia)
AD-MSC 1102	Adipose-derived mesenchymal stromal cells	no	Institute of Cytology RAS, St. Petersburg, Russia
AD-MSC 2001	Adipose-derived mesenchymal stromal cells	no	Institute of Cytology RAS, St. Petersburg, Russia

**Table 2:** Properties of the qPCR primers used.

Gene Symbol	RefSeq Accession Number	Encoded Protein/RNA	Forward Primer Reverse Primer Fluorescent Probe (If Used) 5′-3′	Amplicon Length	Ref.
***EIF2B1***	NM_001414.4	Translation initiation factor 2B subunit alpha (EIF2B1)	GCCATGGACGACAAGGAGTT ACCCTGGATTGTCTCCCCTT	135	[48]
***POP4***	NM_006627.3	RibonucleaseP/ MRP subunit (POP4)	ACCAGAGCAGCAGAGATACA ATCTGCCTTTAAGAGCTTGGC	133	[48]
***YWHAZ***	NM_003406.4	Tyrosine 3-monooxygenase/ tryptophan 5-monooxygenase activation protein, zeta polypeptide, KCIP-1	CGAAGCTGAAGCAGGAGAAG TTTGTGGGACAGCATGGATG	110	[80]
***EGFR/HER1***	NM_201284.2 NM_201283.2 NM_001346899.2 NM_001346897.2 NM_201282.2 NM_001346900.2 NM_001346898.2 NM_005228.5	Epidermal growth factor receptor	GGAGAACTGCCAGAAACTGACC GCCTGCAGCACACTGGTTG	106	[81]
***HER2***	NM_001382782.1 NM_001289936.2 NM_001005862.3 NM_001289938.2 NM_001382783.1 NM_001382787.1 NM_001382784.1 NM_001382786.1 NM_001382789.1 NM_001382788.1 NM_001382785.1 NM_004448.4 NM_001289937.2 NM_001382796.1 NM_001382798.1 NM_001382800.1 NM_001382797.1 NM_001382805.1 NM_001382792.1 NM_001382793.1 NM_001382803.1 NM_001382794.1 NM_001382795.1 NM_001382801.1 NM_001382790.1 NM_001382806.1 NM_001382802.1 NM_001382799.1 NM_001382791.1 NM_001382804.1	Human epidermal growth factor receptor 2	ACAACCAAGTGAGGCAGGTC GTATTGTTCAGCGGGTCTCC	115	THIS ARTICLE
***HER3***	NM_001005915.1 NM_001982.4	Human epidermal growth factor receptor 3	GGTGATGGGGAACCTTGAGA AGCCTGTCACTTCTCGAATCC /FAM/-TGCTCACGGGACACAATGCCGACC-/BHQ1	83	THIS ARTICLE
***HER4***	NM_001042599.1 NM_005235.3	Human epidermal growth factor receptor 4	GTTCAGGATGTGGACGTTGC GTTCTGCACACACACCGTCCTT	99	THIS ARTICLE

## Data Availability

The original contributions presented in this study are included in this article/Supplementary Materials. Further inquiries can be directed to the corresponding authors.

## Supplementary Materials

The following supporting information can be downloaded at: https://www.mdpi.com/article/10.3390/ijms26157201/s1.

## Abbreviations

The following abbreviations are used in this manuscript:

EGF	epidermal growth factor
TGF-α	transforming growth factor -α
HB-EGF	heparin-binding EGF-like growth factor
HER1-4	human EGF receptor 1-4
EGFR	EGF receptor
MSCs	mesenchymal stromal cells
RT-qPCR	quantitative reverse transcription polymerase chain reaction
