# Supplementary material for: Human Mesenchymal Stromal Cells Derived from Different Tissues Show Similar Profiles of c-ErbB Receptor Family Expression at the mRNA and Protein Levels
Source: Int J Mol Sci. 2025 Jul 25;26(15):7201. doi: 10.3390/ijms26157201 (PMC12347453; doi:10.3390/ijms26157201)
Supplement: Supplementary file 1 [file ijms-26-07201-s001.zip › Figure S1.pdf]

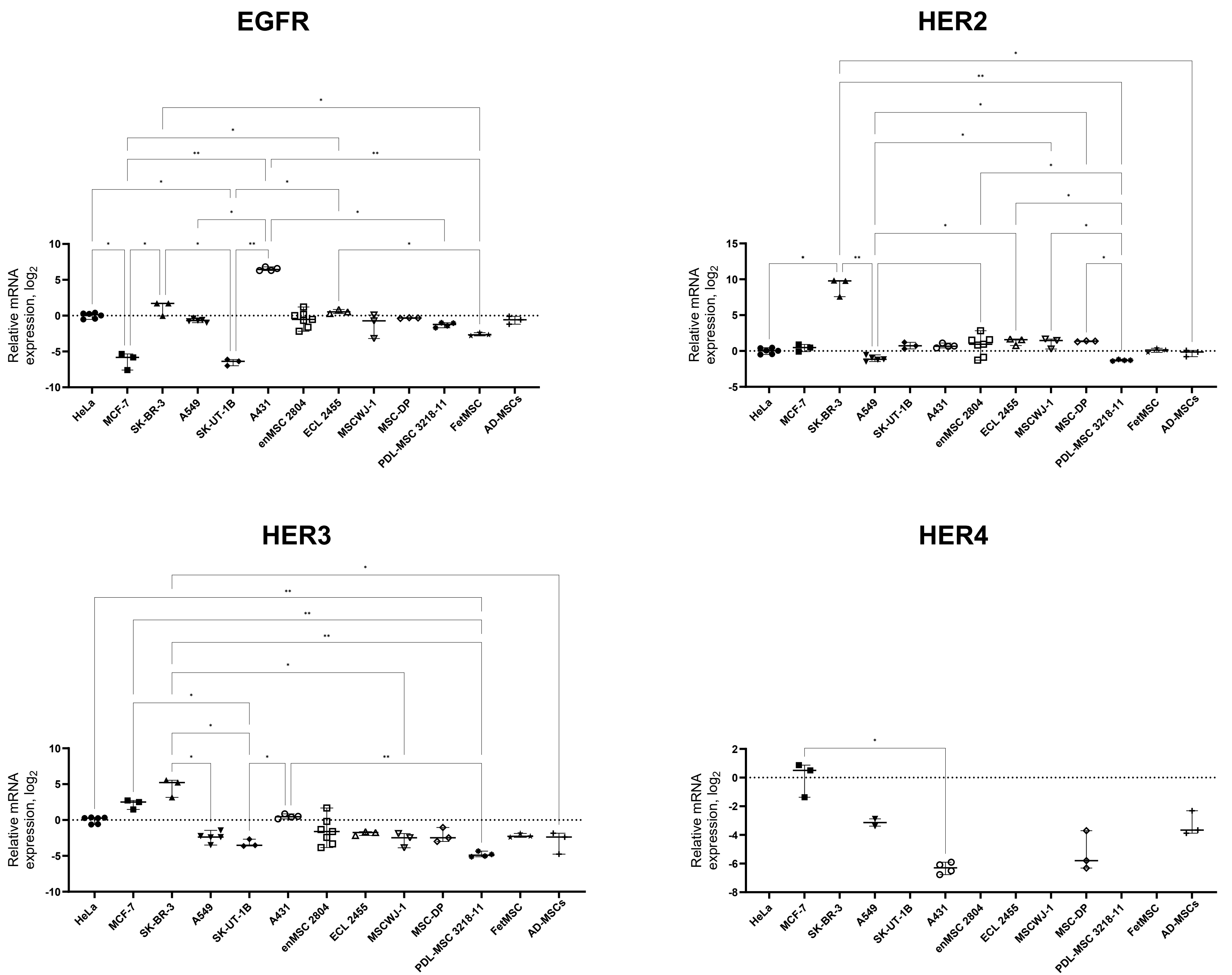

Figure S1: Relative expression of EGFR, HER2, HER3, HER4 genes in human malignant and mesenchymal stromal cells, derived from different tissues.
