# Supplementary material for: Human Mesenchymal Stromal Cells Derived from Different Tissues Show Similar Profiles of c-ErbB Receptor Family Expression at the mRNA and Protein Levels
Source: Int J Mol Sci. 2025 Jul 25;26(15):7201. doi: 10.3390/ijms26157201 (PMC12347453; doi:10.3390/ijms26157201)
Supplement: Supplementary file 1 [file ijms-26-07201-s001.zip › Figure S2.pdf]

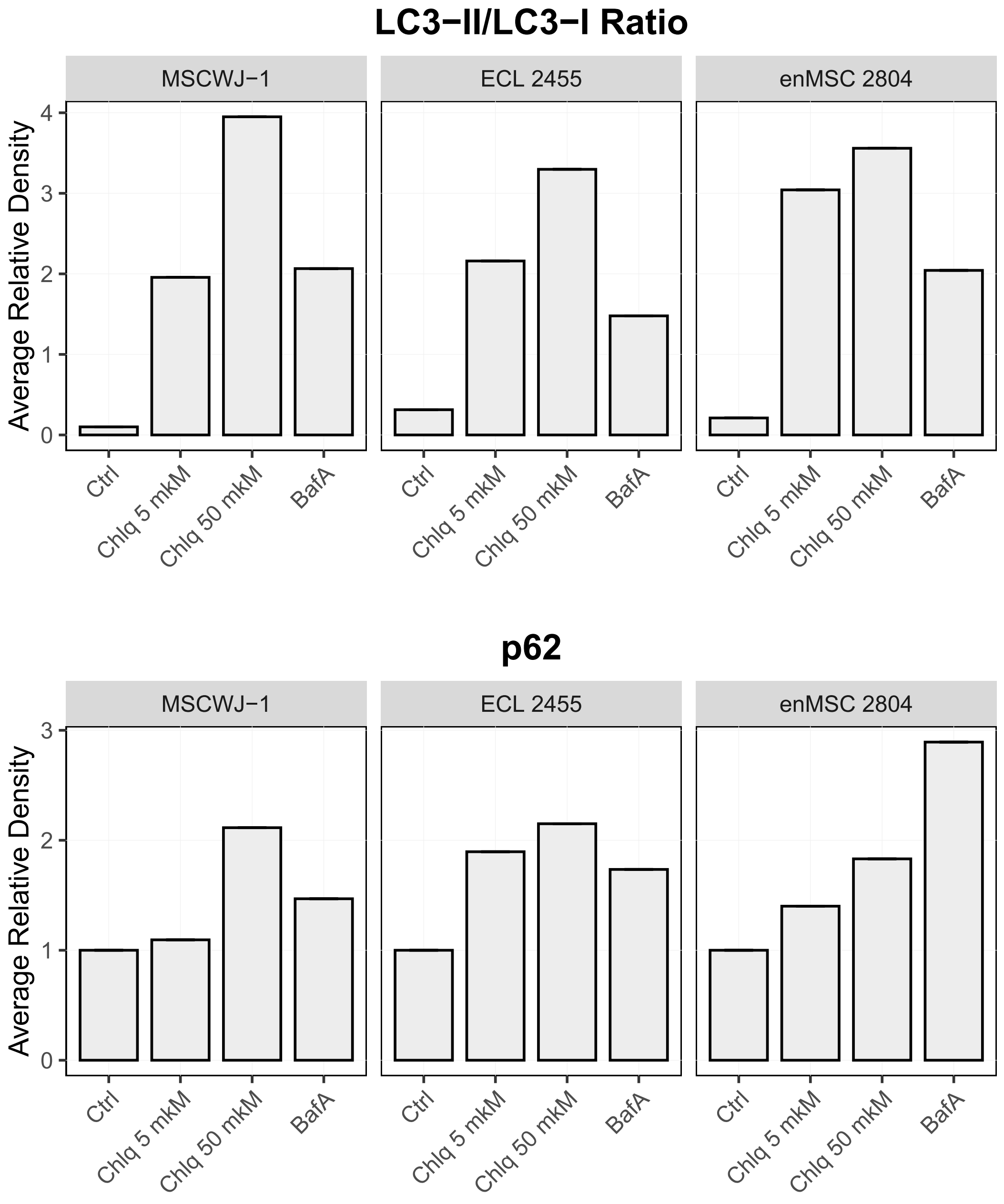

Figure S2: Densitometry of LC3A/B and p62. The Western blots from Figure 3 were quantified and normalized against each line control cells (Ctrl). The densitometry data are presented as the means.
