# Supplementary material for: Human Mesenchymal Stromal Cells Derived from Different Tissues Show Similar Profiles of c-ErbB Receptor Family Expression at the mRNA and Protein Levels
Source: Int J Mol Sci. 2025 Jul 25;26(15):7201. doi: 10.3390/ijms26157201 (PMC12347453; doi:10.3390/ijms26157201)

Original blots for Fig.2

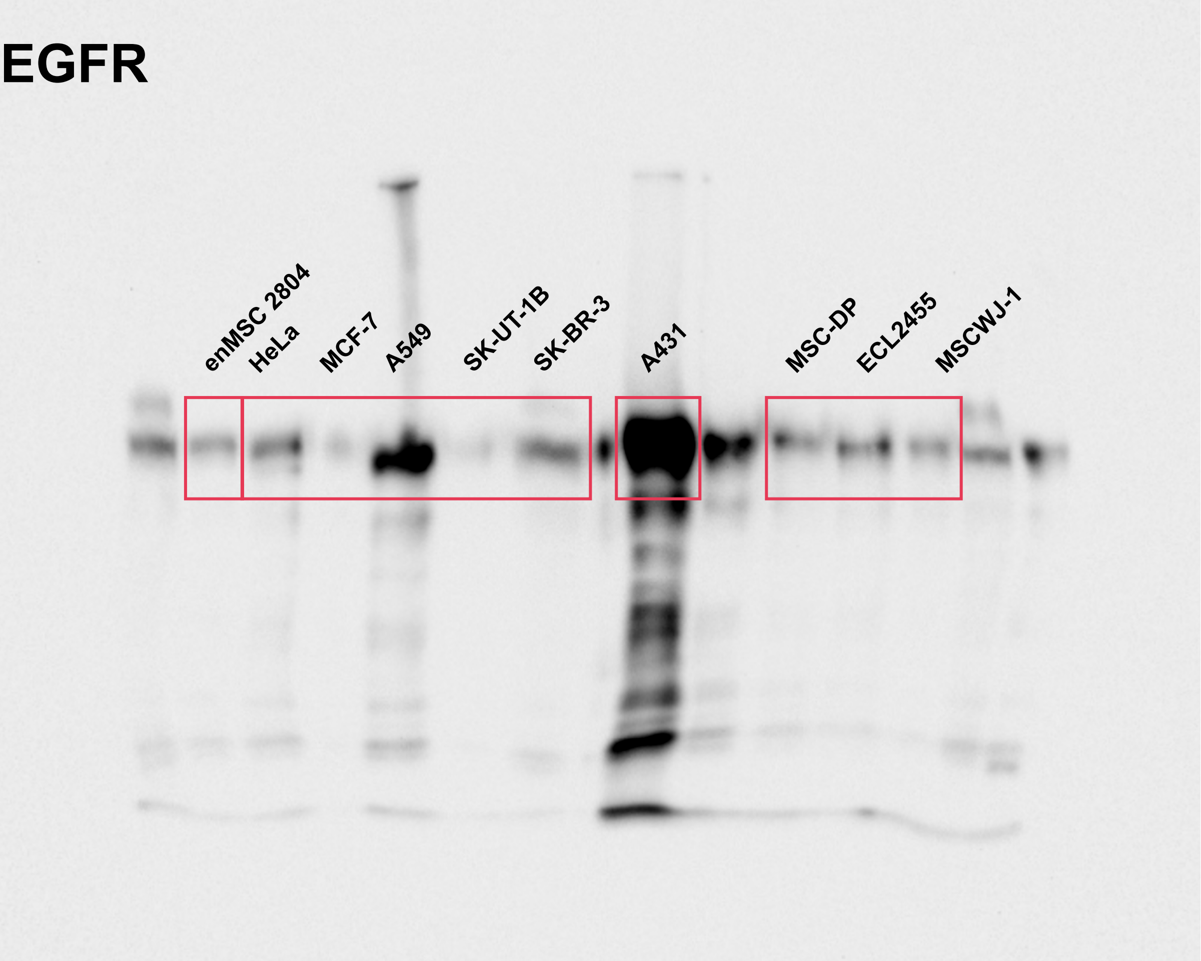

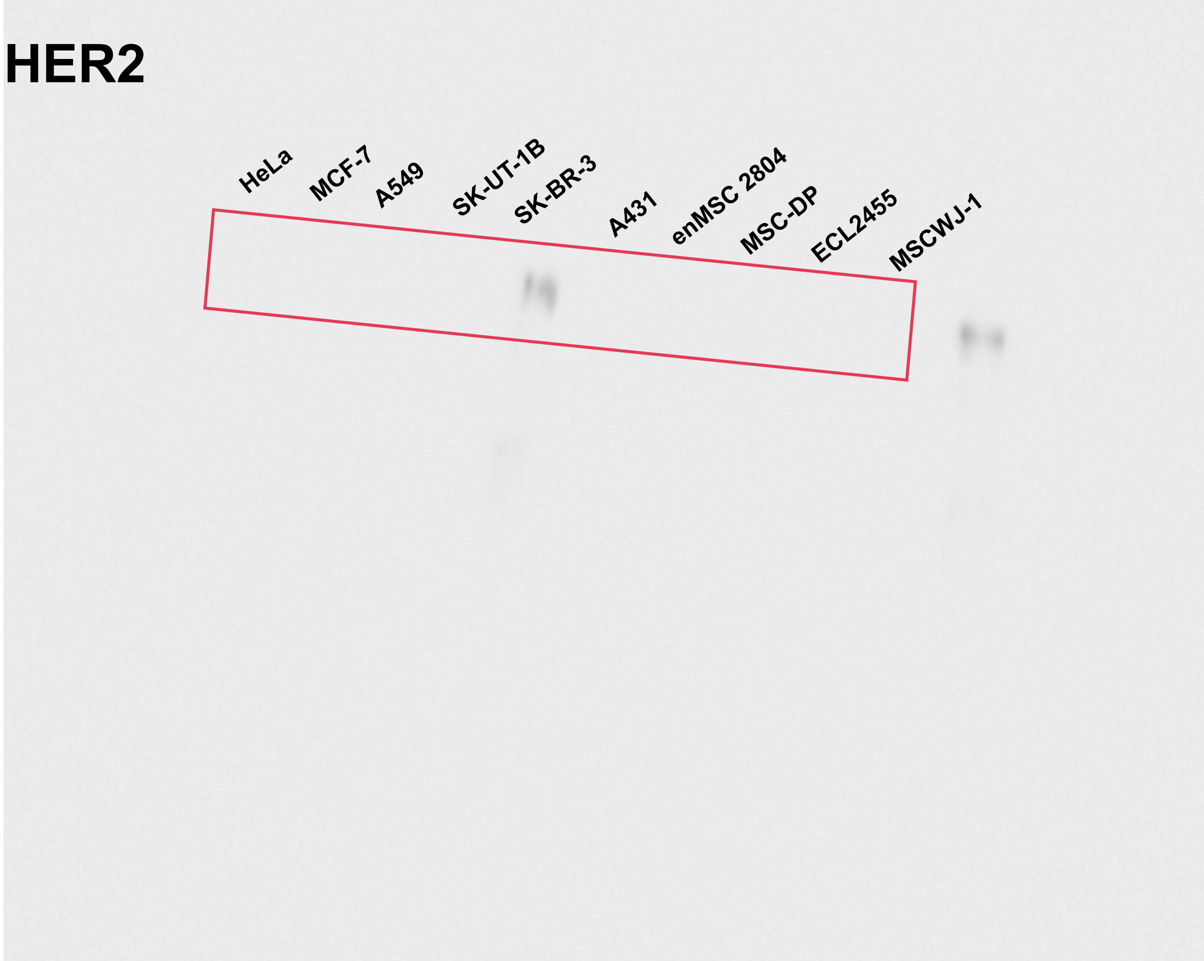

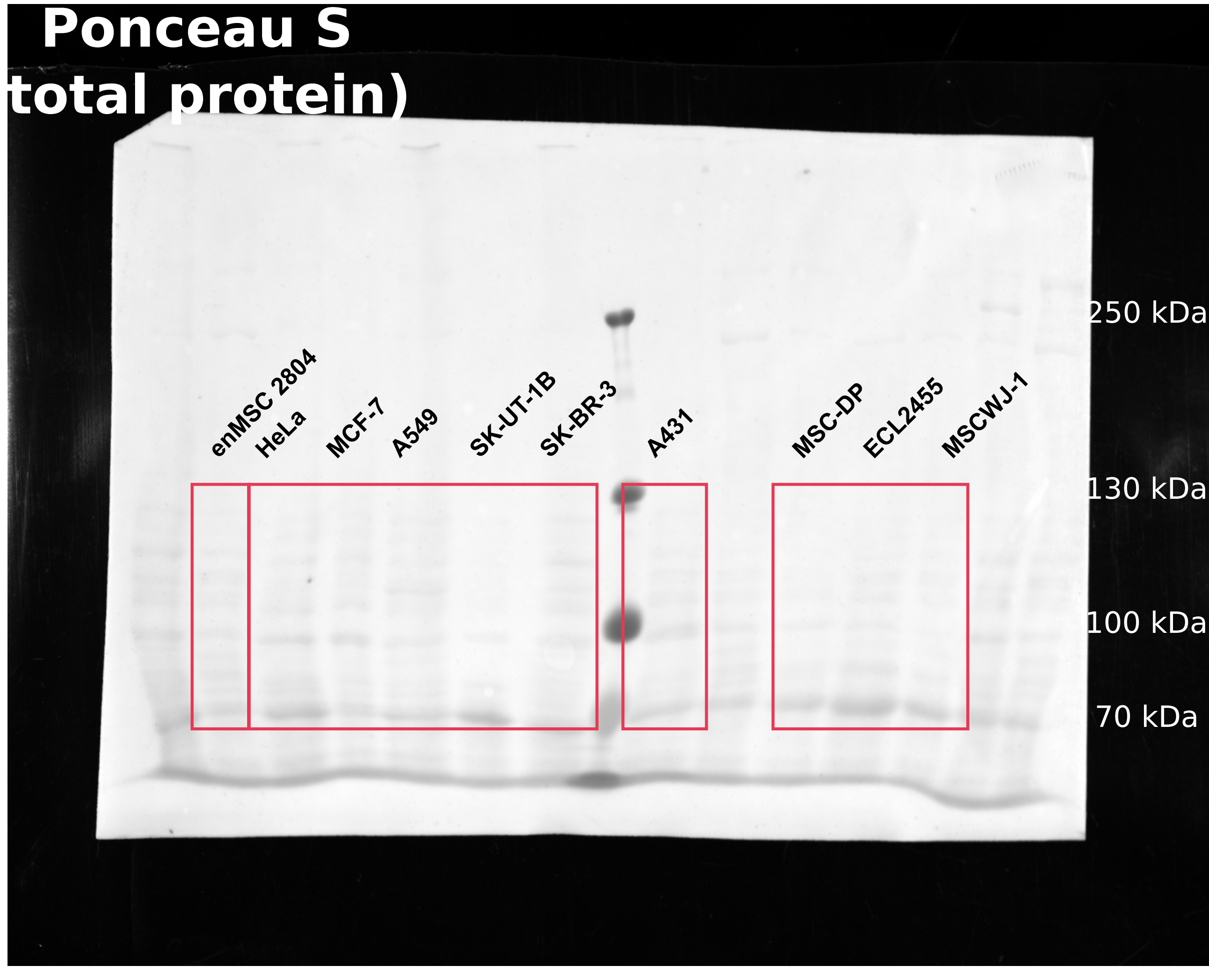

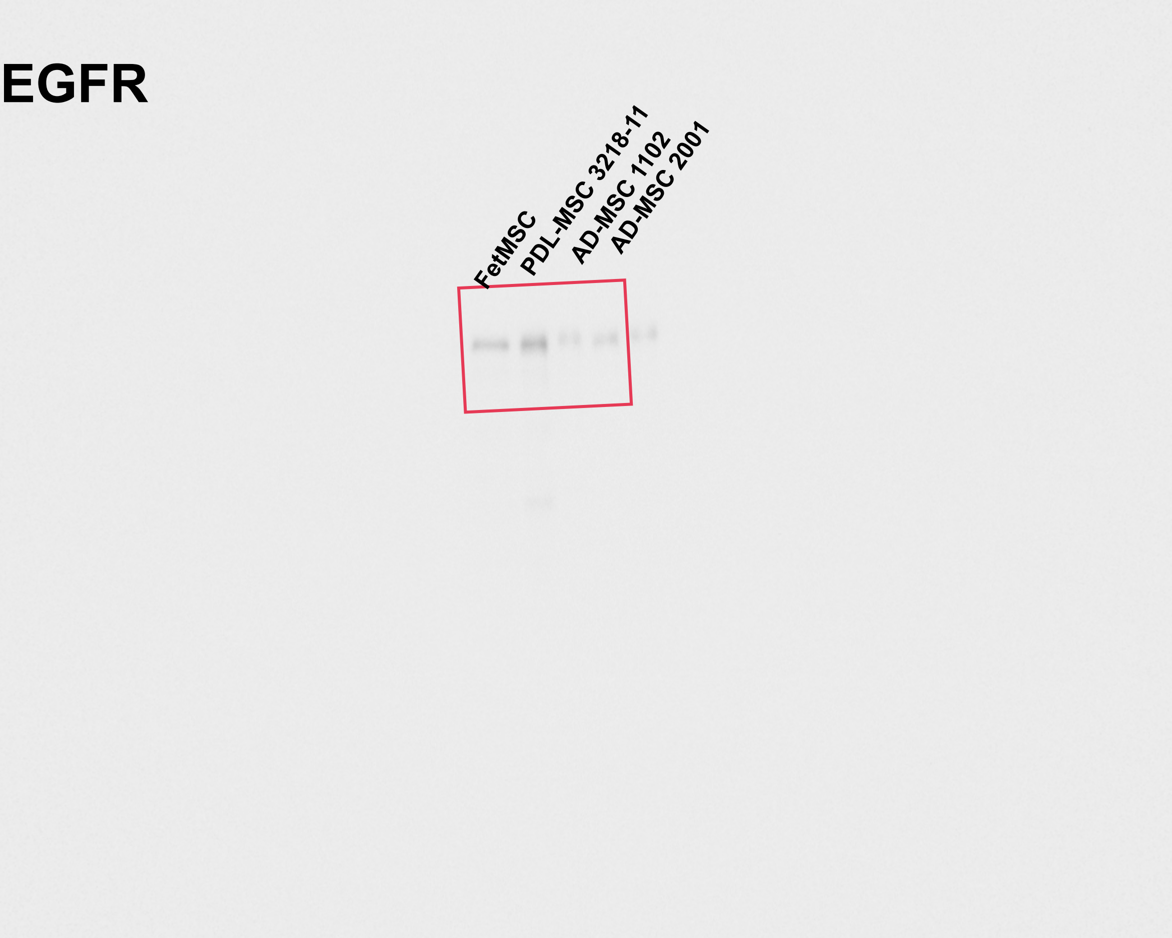

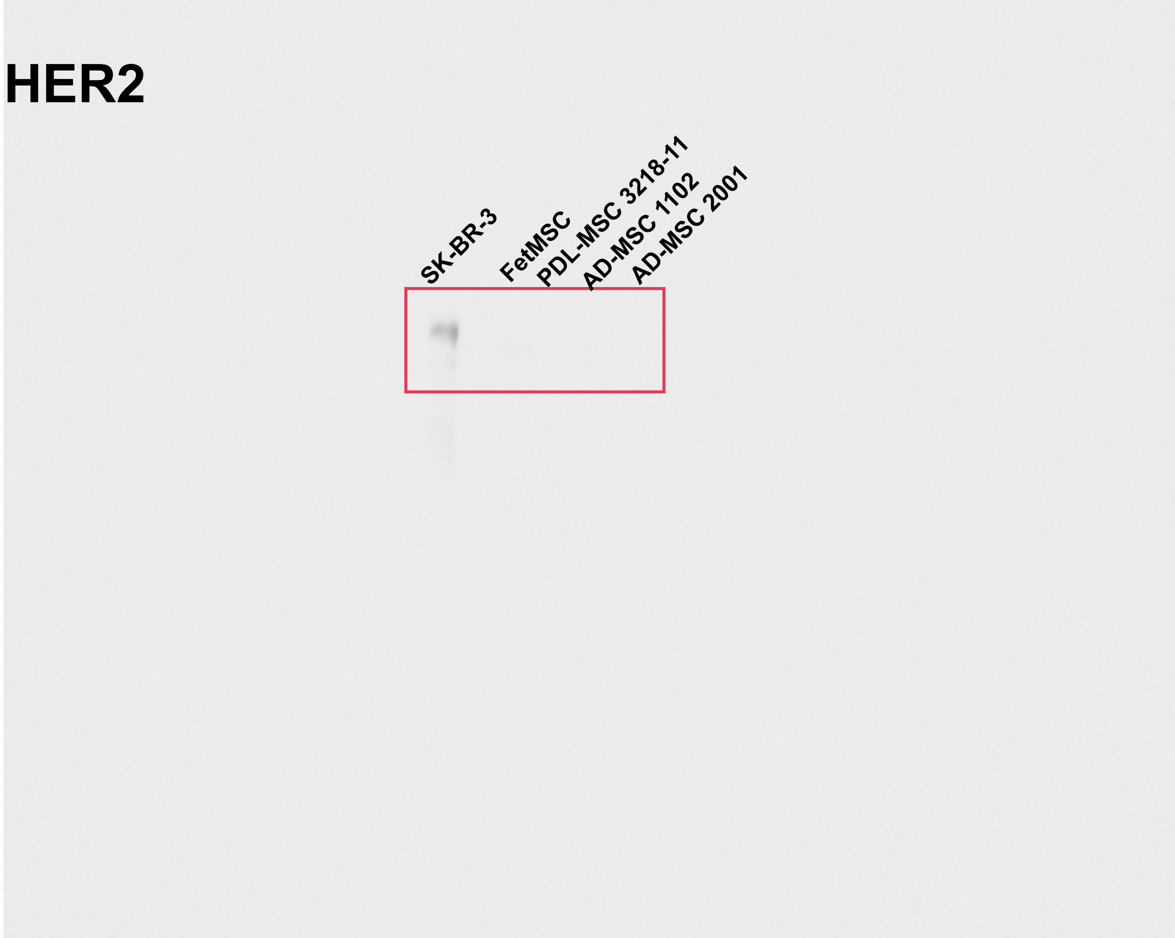

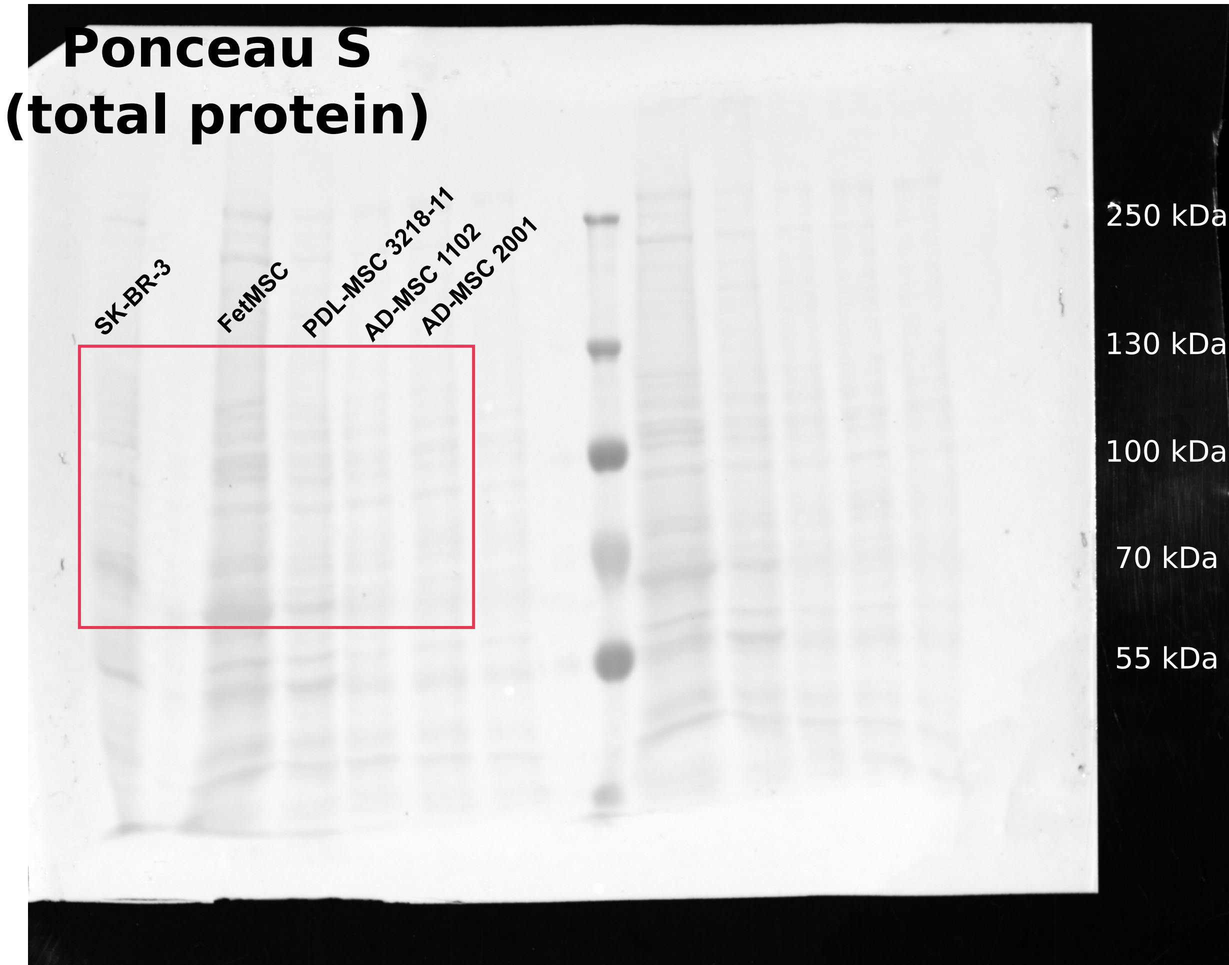

Original blots for Fig.3

EGFR

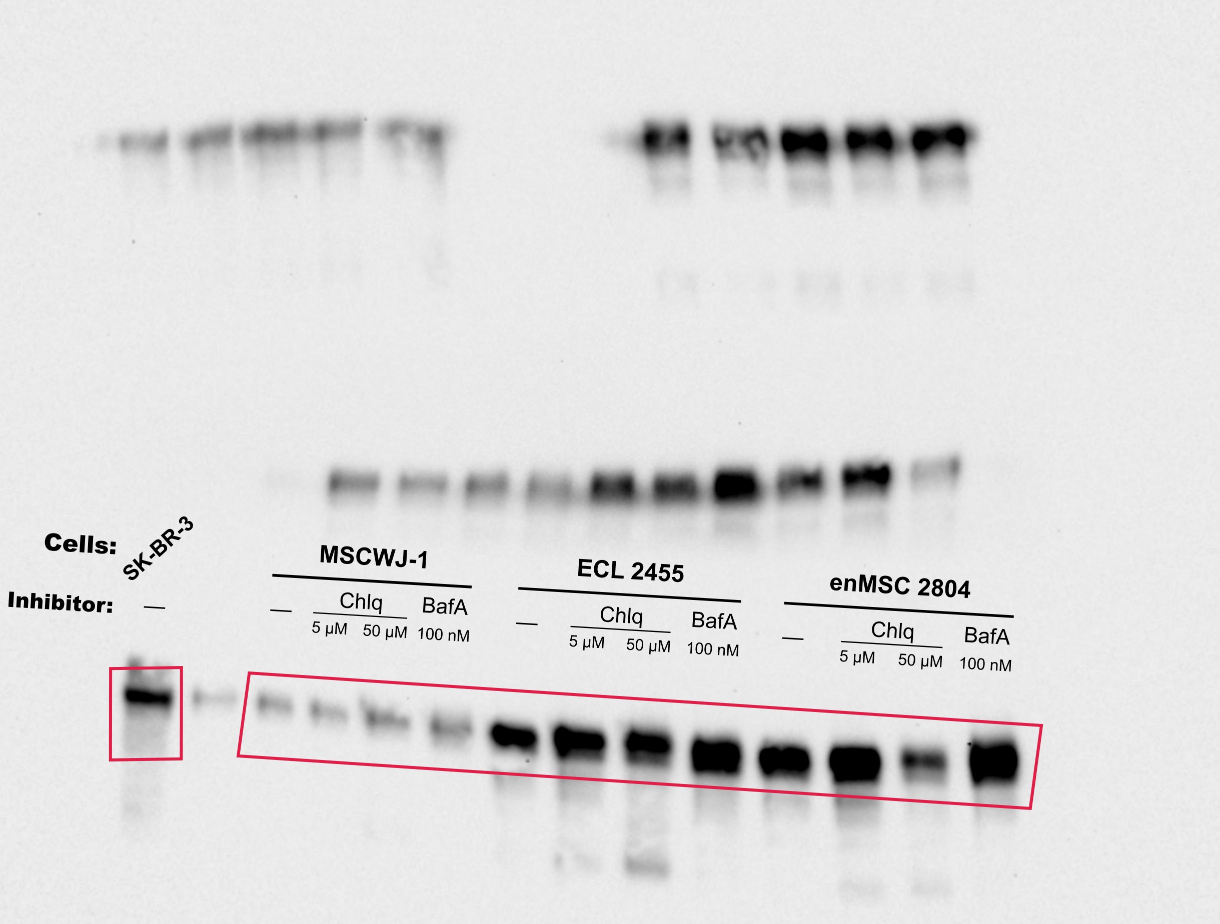

HER2

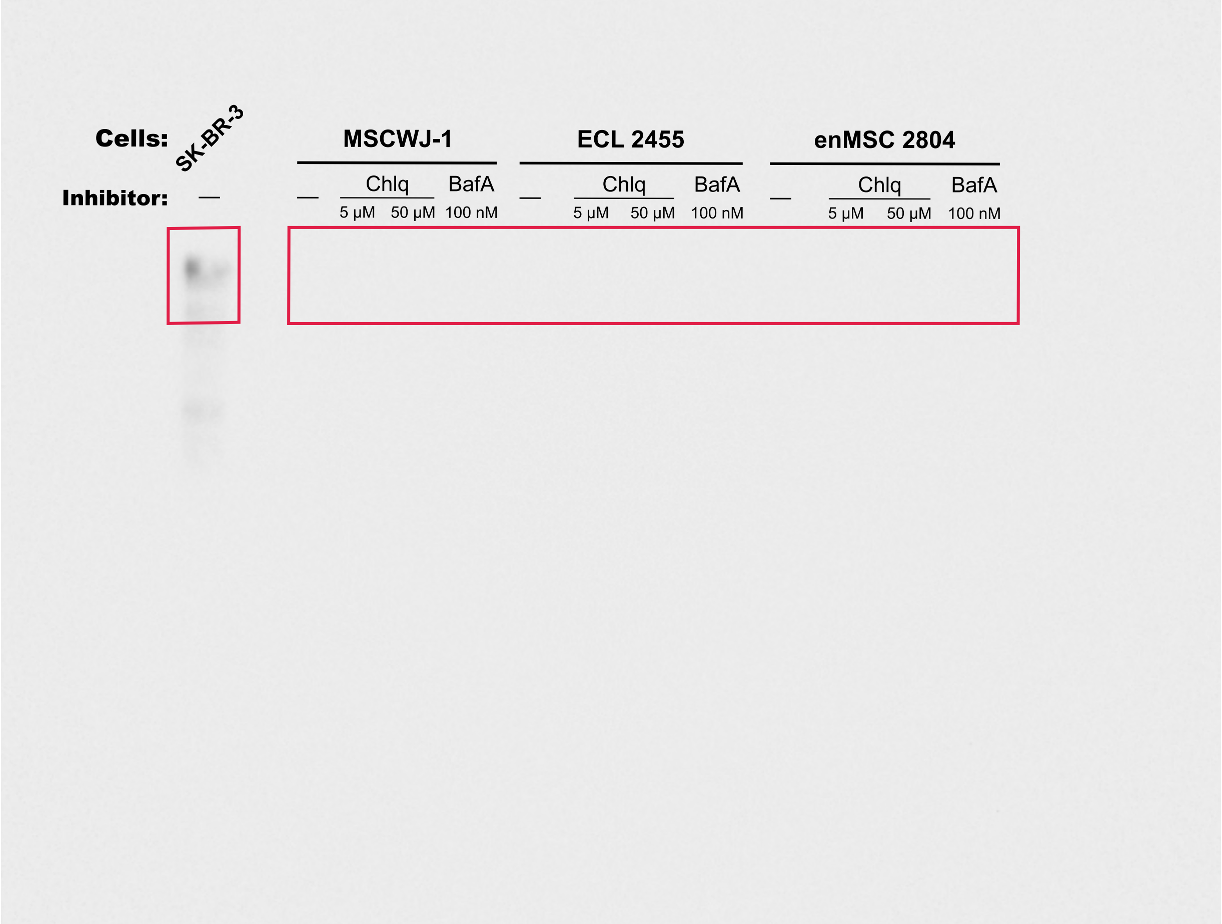

α-tubulin

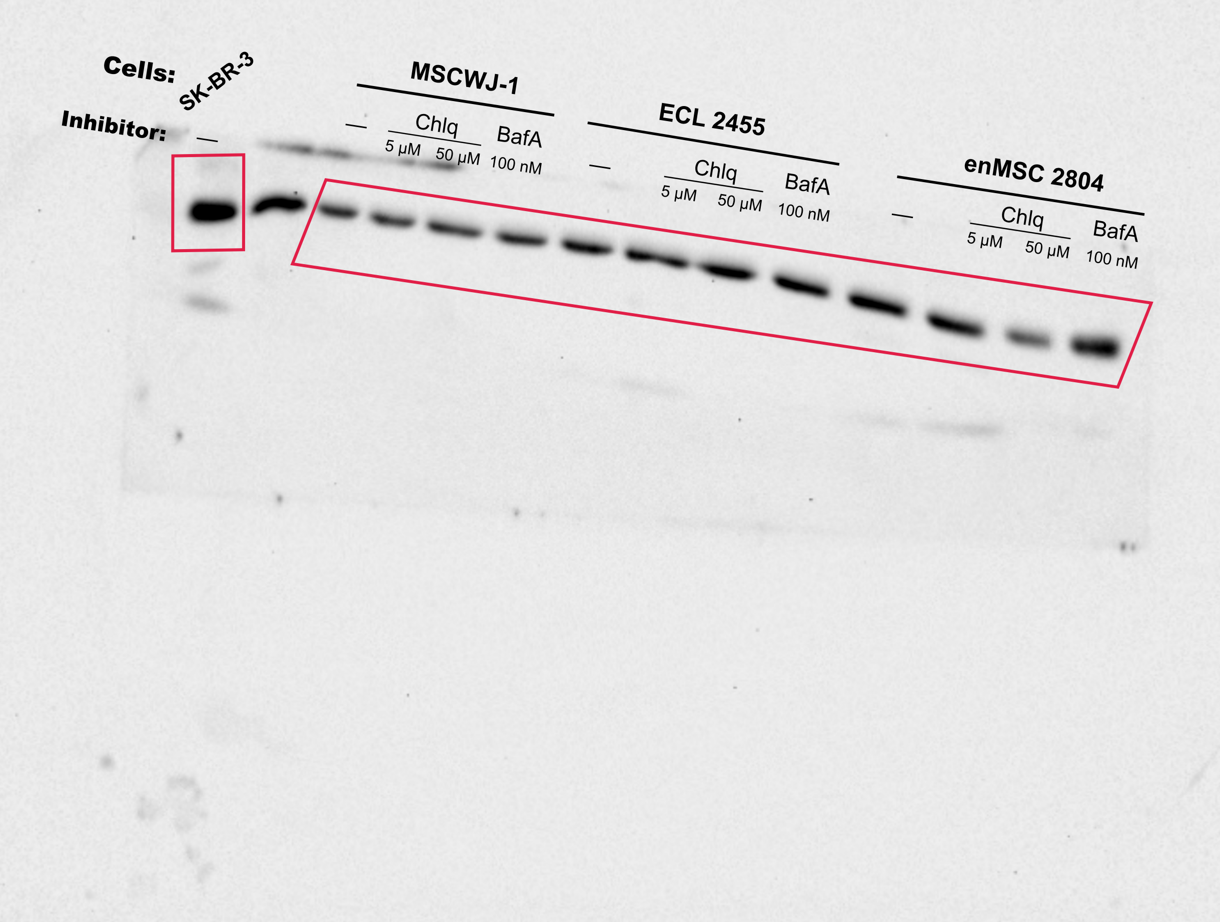

Ponceau S (total protein)

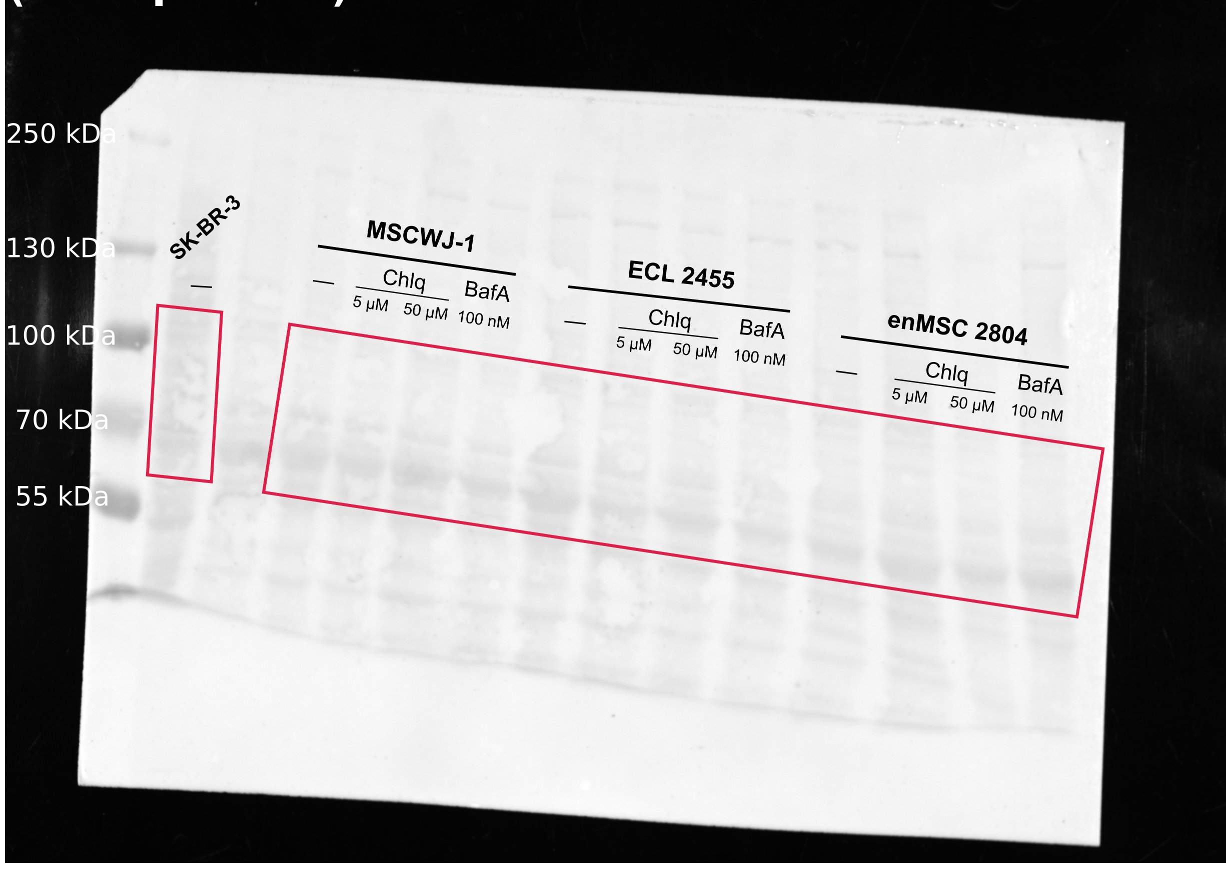

p62

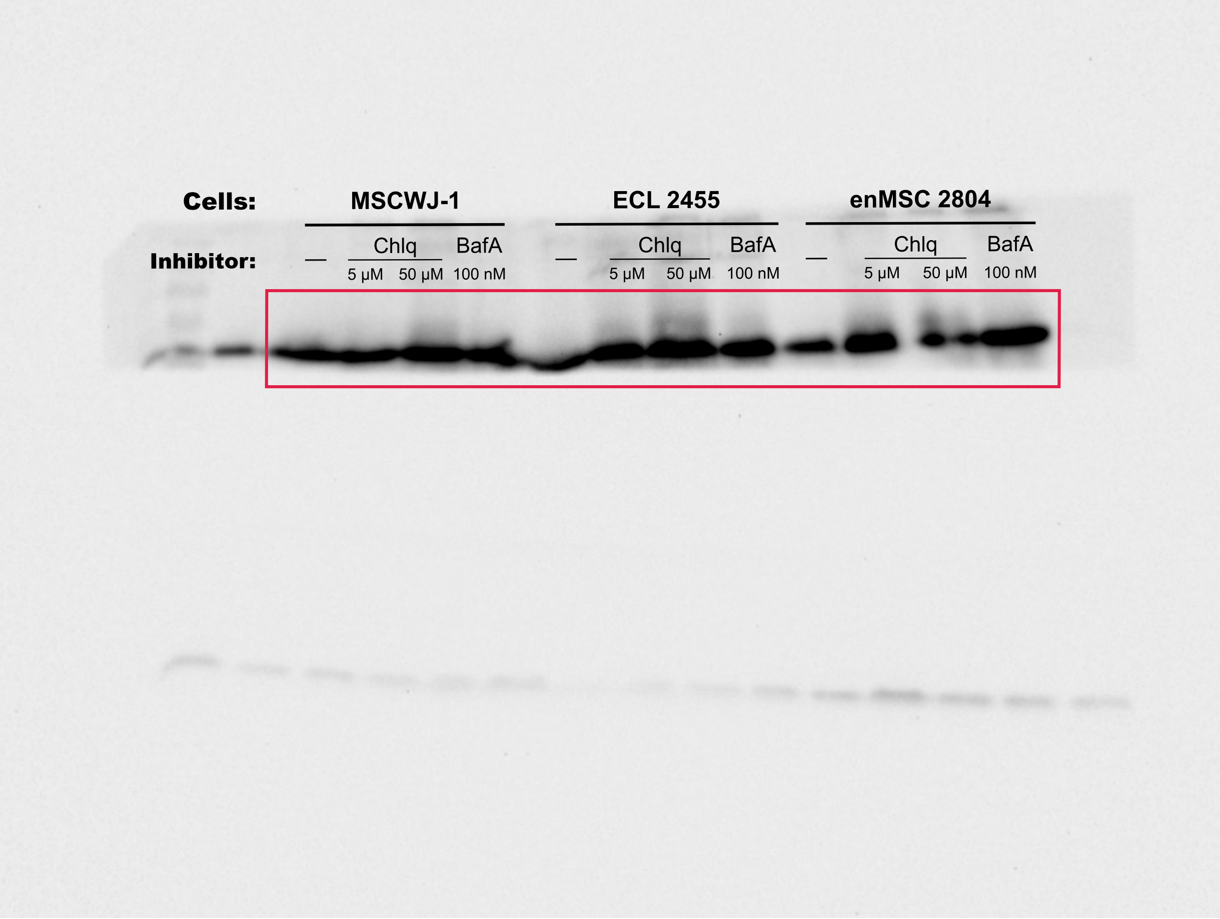

LC3A/B

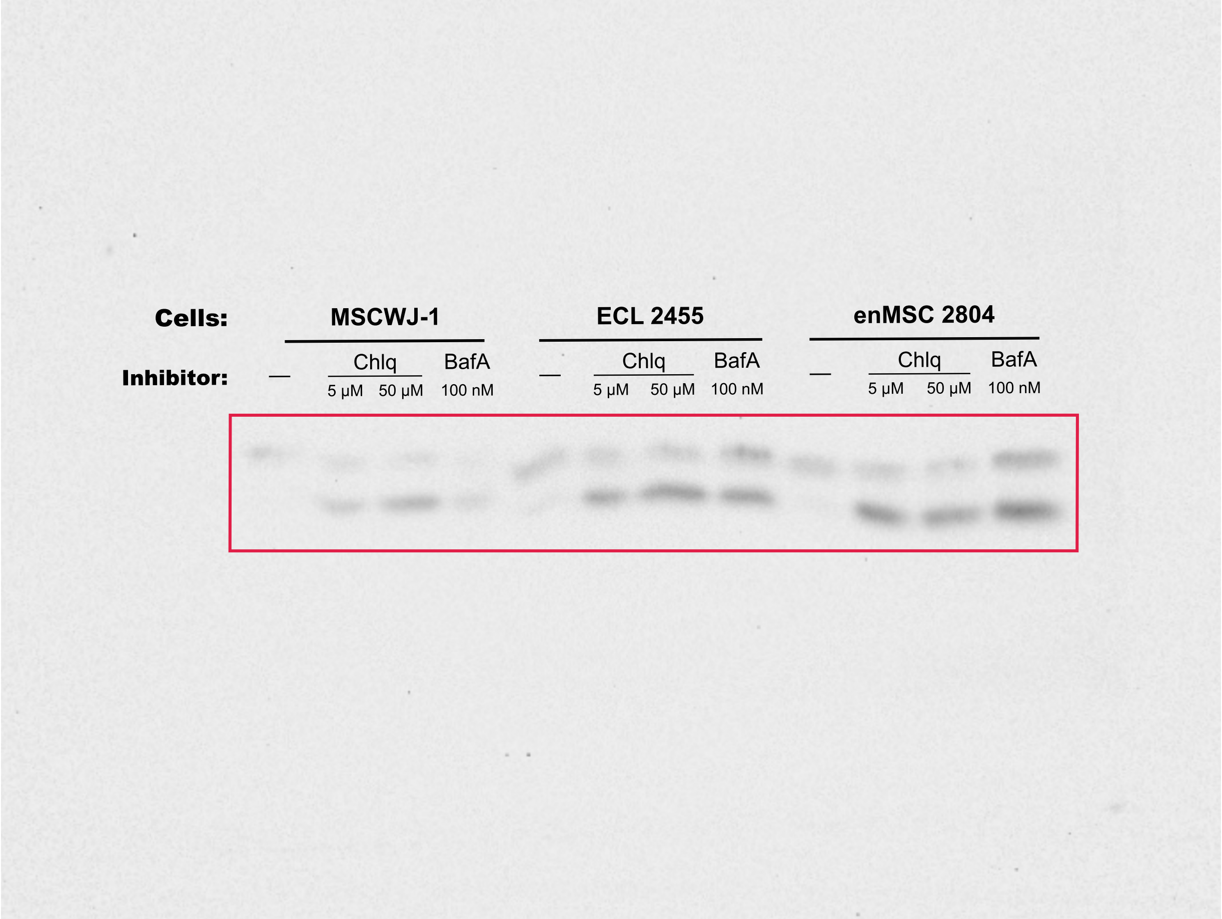

Supplement: Supplementary file 1 [file ijms-26-07201-s001.zip › OriginalBlots.pdf]
